# First Molecular Detection of Piroplasm Infection in Pet Dogs from Gansu, China

**DOI:** 10.3389/fmicb.2017.01029

**Published:** 2017-06-07

**Authors:** Qingli Niu, Jifei Yang, Zhijie Liu, Shandian Gao, Yuping Pan, Guiquan Guan, Yuefeng Chu, Guangyuan Liu, Jianxun Luo, Hong Yin

**Affiliations:** ^1^State Key Laboratory of Veterinary Etiological Biology, Key Laboratory of Veterinary Parasitology of Gansu Province, Lanzhou Veterinary Research Institute, Chinese Academy of Agricultural ScienceLanzhou, China; ^2^Jiangsu Co-innovation Center for Prevention and Control of Important Animal Infectious Diseases and ZoonosesYangzhou, China

**Keywords:** pet dog, piroplasm, detection, Gansu, infection

## Abstract

Babesiosis, the hemolytic disease caused by *Babesia*, which is a tick-transmitted obligate intraerythrocytic protozoan parasite. This disease is responsible for significant mortality and morbidity rates and enormous economic losses to the livestock industry in tropical and subtropical regions worldwide. In this study, blood samples were collected from 141 pet dogs from Gansu, China, and analyzed for *Babesia* or *Theileria* spp. infection using specific PCR and sequencing based on 18S rRNA gene fragments. The results indicated that 18S rRNA gene sequences from 11 samples were similar to the 18S rRNA gene sequences in *Babesia canis vogeli* (2) and *Theileria sinensis* (9). The total infected rates of *B. canis vogeli* and *T. sinensis* were 1.4% (2/141) and 6.4% (9/141), respectively. This represents the first molecular report of *T. sinensis* in dogs worldwide and of *B. canis vogeli* in dogs from Gansu province of China. Furthermore, the finding of *T. sinensis* in dogs may represent the common infection of this parasite occurring in Gansu.

## Introduction

Babesiosis is a haemoparasitic disease, caused by the intraerythrocytic multiplication of protozoa of the genus *Babesia* and transmitted by ticks. It is a frequent infection of domestic and wild animals worldwide, including humans (Telford et al., 1993; Bush et al., 2001). The clinical symptoms of babesiosis are due to the repeated asexual rounds of multiplication of parasites inside of host erythrocytes, and are usually characterized by fever, depression, hemolytic anemia, hemoglobinuria, icterus, finally resulting in death in severe cases if not treated. The prevalence of babesiosis correlates with the geographic distribution and activity of vector ticks. Furthermore, environmental conditions changing, including global warming, favors tick survival and reproduction, which correlate with a significant increase in the abundance of ticks (Slenning, 2010). This disease can be responsible of great direct and indirect economic losses, due to the death of the animals, a reduction in the production or restriction in animal movements (McCosker, 1981).

Canine babesiosis is a common and clinically significant emerging hemoprotozoan infection of domestic dogs and wild canids geographically widespread by tick borne pathogen *Babesia* (Víchová et al., 2016). The clinical characteristics include fever, haemolytic anemia, thrombocytopenia, and vomiting. The earliest descriptions of intraerythrocytic parasites in dogs were in Africa in 1896, and the first documented case of canine babesiosis was in the United States in 1934 (Adaszek and Winiarczyk, 2008). Recently, six main *Babesia* species responsible for canine babesiosis have been reported in the world, including the large form of *B. canis* (three distinct subspecies *B. canis canis*, *B. canis rossi*, and *B. canis vogeli*) and the small form of *B. gibsoni*, *B. conradae*, and *B. vulpes* (Zahler et al., 2000; Boozer and Macintire, 2003; Irwin, 2009, 2010; Matijatko et al., 2012; Berzina et al., 2013; Kamani et al., 2013; Baneth et al., 2015). Moreover, two large *Babesia* spp. have been reported, one is distributed across North Carolina, New Jersey, New York, and Texas (Birkenheuer et al., 2004; Holman et al., 2009; Sikorski et al., 2010), and another one was only reported in a dog from Great Britain (Holm et al., 2006). Arthropod vectors of these two *Babesia* species have not been identified.

Little information is available on the prevalence of piroplasmosis in dogs in Gansu province, China. Therefore, the aim of the present study was to investigate the occurrence of *Babesia* and *Theileria* spp. infections in pet dogs from Gansu province of China.

## Materials and Methods

### Animals and Sample Collection

A total of 141 blood samples were collected from randomly selected pet dogs (84 males and 57 females) of different breeds in a pet clinic. Venous blood samples were collected into EDTA tubes between March 2015 and March 2016. The ages of the dogs were between 2 months and 18 years. One hundred of the pet dogs were without clinical symptom, and 41 animals presented with clinical signs: fever, pale mucous, vomiting, chough, and thrombocytopenia, and thus were suspected to have haemoparasite infection.

### Genomic DNA Extraction, PCR Amplification and Sequencing

Genomic DNA was isolated from 300 μl of each blood sample using a QIAamp DNA Blood Mini Kit (Gentra, United States) according to the manufacturer’s instructions. The DNA samples were stored at -20°C until further use. Genomic DNA from *Babesia* sp. Lintan (*Babesia* cf. *motais*) and *T. ovis* was used as the positive control and distilled water was used as the blank control.

For the molecular detection and identification of piroplasm parasites at the molecular level, PCR amplification of the 18S rRNA gene was performed with the genomic DNA of all samples. A set of forward and reverse primers (Piro1-S: 5′-CTTGACGGTAGGGTATTGGC-3′, Piro3-AS: 5′-CCTTCCTTTAAGTGATAAGGTTCAC-3′) was used to amplify a gene fragment of 1400 bp (Yang et al., 2014), after which a nested-PCR was performed on primary PCR products with internal primers (PIRO-A: 5′-ATAACCGTGCTAATTGTAGG-3′ and PIRO-B: 5′-TGTTATTTCTTGTCACTACC-3′) to amplify a gene fragment of 406-421 bp (Olmeda et al., 1997). The PCR amplified conditions of the 18S rRNA gene were previously described by Yang et al. (2014). All 114 samples of secondary PCR products were subjected to electrophoresis on 1.5% agarose gels treated with GoldView I nucleotide stain (Solarbio) and visualized under UV illumination for the expected size of amplified fragments by comparison to molecular weight marker.

The positive PCR products with 18S rRNA gene fragments of 11 isolates were purified using a MiniBEST DNA Fragment Purification Kit (TaKaRa), cloned into a pGEM-T Easy Vector System (Promega), and then transformed into *Escherichia coli* JM109 Chemically Competent Cells (TaKaRa) according to the manufacturers’ instructions. Colonies were selected by direct colony PCR using vector primers. Ten sub-colonies from each sample were selected for sequencing.

The 11 partial 18S rRNA sequences obtained were subjected to a blast search on the NCBI website^1^ using the BLASTn program and deposited in GenBank under accession nos. KY608898-KY608908. Multiple sequence alignments were analyzed using Clustal W 2.0.12 software. A phylogenetic tree was constructed with the sequences obtained in this study and sequences of the 18S rRNA genes of the main *Babesia* and *Theileria* species available in GenBank using Neighbor-Joining in MEGA 7 software (Kumar et al., 2016).

### Ethics Statement

This study was approved by the Animal Ethics Committee of the Lanzhou Veterinary Research Institute, Chinese Academy of Agricultural Sciences (No. LVRIAEC2013-010). All pet dogs were handled in accordance with the Animal Ethics Procedures and Guidelines of the People’s Republic of China.

## Results and Discussion

Vector-borne infections in dogs have been increasing worldwide. However, few studies have been performed on piroplasm in dogs in Gansu. In 2015, there was a report investigating prevalence of tick-borne pathogens in 10 provinces of China, including Gansu province, but no samples indicated *Babesia* or *Theileria* species infections in dogs from Gansu (Xu et al., 2015).

In our study, out of the 141 dogs sampled, 11 positive samples for piroplasm infections were found, including 2 (1.4%) infected with *B. canis vogeli* and 9 (6.4%) infected with *T. sinensis*. Two pet dogs (one male, Shiba Inu and one female, mixed-breed) infected with *B. canis vogeli* were both 2 months old and showed the clinical symptom of fever. Studies have revealed that young animals are susceptible to *B. canis vogeli* infection and their clinical presentations are more severe (Greene, 2011). At present, *B. canis vogeli* and *B. gibsoni* have been reported to infect dogs in Jiangxi, Fujian, Anhui, Jiangsu, Chongqing, Guangdong, Guangxi, Hainan, Zhejiang, Shanghai, and Shandong provinces (Shen et al., 1997; Wei et al., 2012; Cao et al., 2015). The transmission vector for *B. canis vogeli* is *Rhipicephalus sanguineus*, a tick that is distributed in 15 provinces of China, including Gansu (Teng and Jiang, 1991). No *B. gibsoni* infections were found in pet dogs in our study. *T. sinensis*, which normally infects cattle, was found to infect two pet dogs (male, 6 years old, asymptomatic) belonging to the Bichon Frise breed; 2 Nisos dogs (male, one was 2 months old and one was 5 months old) with the clinical feature of fever; one Husky dog (male, 7 months old) with a cough; and four mixed-breed dogs (three males, two of them are 3 months old and another one was 8 years old; one female, 4 months old) with the clinical features of fever, vomiting and cough. Several studies based on molecular methods have revealed that dogs could be sporadically infected by the genus *Theileria*, including *Theileria equi* (Beck et al., 2009), *T. ovis* (Takeet et al., 2017), *T. annulata* (Criado et al., 2006; Aktas et al., 2015) and *T. orientalis* (Xu et al., 2015), from Croatia, Spain, Turkey, Iran, and China. These parasites are usually found in horses, sheep and cattle. *T. sinensis* is distributed only in Gansu Province China and is transmitted by *Haemaphysalis qinghaiensis* (Zhao et al., 2017). In our study, records from dogs infected with *B. canis vogeli* and *T. sinensis* provided by veterinarians from clinics indicated that these pet dogs spent most of their time indoors and they had limited exposure to the outside environment with active *R. sanguineus* and *H. qinghaiensis* ticks. Thus, how these dogs were exposed to *B. canis vogeli* and *T. sinensis* requires further study. Possibilities of blood transfusion and vertical transmission might be existed.

**FIGURE 1:**
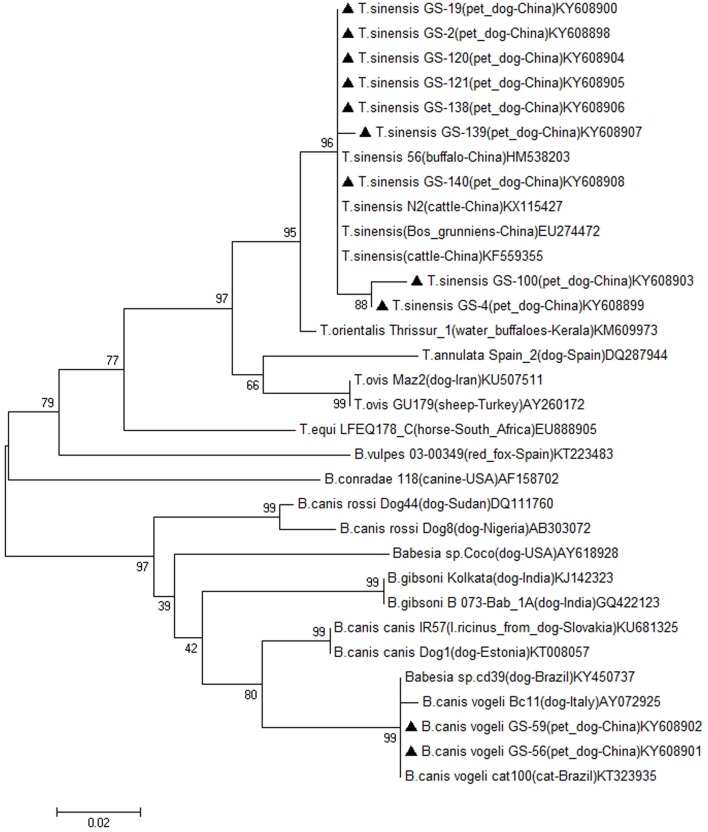
Phylogenetic tree of the nucleotidic sequences of *Babesia* and *Theileria* spp. 18S rRNA obtained from pet dogs in this study and deposited in GenBank from different piroplasm species, the isolate, host, countries and accession numbers are shown after species name. The 18S rRNA sequences obtained in this study were indicated with bold triangle. The tree was inferred using the neighbor joining method of MEGA7, bootstrap values are shown at each branch point. Numbers above the branch demonstrate bootstrap support from 1000 replications. All sites of the alignment containing insertions–deletions, missing data were eliminated from the analysis (option “complete deletion”).

A phylogenetic tree, based on 18S rRNA sequences (*n* = 11) determined in the present study and sequences of this gene deposited in GenBank from different species, was constructed by Neighbor-Joining method using the software MEGA 7 (**Figure 1**). In general, the tree indicated that the 18S rRNA sequences formed two clades, including *Theileria* spp. clade and *Babesia* spp. clade. Nine Gansu isolates formed two sub-branches in the first clade, forming a sister clade with all the *Theileria* spp. 18S rRNA sequences from our study, and formed on the same sub-clade with *T. sinensis*, which normally infected cattle. Two Gansu isolates formed a sub-branch with *B. canis vogeli* isolates from Brazil and Italy. Other canine *Babesia* species formed separated sub-clusters on *Babesia* spp. clade, including *B. canis canis*, *B. gibsoni*, *B. canis rossi* clusters, while *B. vulpesm*, *B. conradae* and *Babesia* sp. Coco formed single clusters, respectively. Taken together, based on the sequences and phylogenetic analysis, the isolates from pet dogs might be infected two piroplasm species, *T. sinensis* and *B. canis vogeli*.

To the best of our knowledge, this is the first report of *B. canis vogeli* in dogs from Gansu province and the first report of *T. sinensis* in dogs in the world. The results of this study provide evidence for the presence of two distinct piroplasm parasites among the canine population from Gansu that had previously not been molecularly documented. It is suggested that increasing piroplasm parasite infections in pet dogs might pose an increased health threat for pet dogs. Dog owners and veterinarians should be better informed on the possibility of infections of canine piroplasm in pet dogs so that appropriate prevention and treatment measures can be adopted.

## Author Contributiions

QN and YP carried out the experiments, including PCR, cloning, sequencing and data analysis. QN drafted the manuscript. JY and YC collected samples, ZL, SG, GG, GL, JL, and HY supervised all work. All authors read and approved the final version of the manuscript.

## Conflict of Interest Statement

The authors declare that the research was conducted in the absence of any commercial or financial relationships that could be construed as a potential conflict of interest.

## Footnotes

1 http://blast.ncbi.nlm.-nih.gov/Blast.cgi
